# Influence of physical and psychosocial work environment throughout life and physical and cognitive capacity in midlife on labor market attachment among older workers: study protocol for a prospective cohort study

**DOI:** 10.1186/s12889-016-3290-8

**Published:** 2016-07-22

**Authors:** Emil Sundstrup, Åse Marie Hansen, Erik Lykke Mortensen, Otto Melchior Poulsen, Thomas Clausen, Reiner Rugulies, Anne Møller, Lars Louis Andersen

**Affiliations:** National Research Centre for the Working Environment, Lersø Parkallé 105, 2100 Copenhagen, Denmark; Department of Public Health, University of Copenhagen, Copenhagen, Denmark; Center for Healthy Aging, University of Copenhagen, Copenhagen, Denmark; Department of Psychology, University of Copenhagen, Copenhagen, Denmark; Department of Occupational Medicine, Holbæk Hospital, Holbæk, Denmark; The Research Unit for General Practice and Section of General Practice, Department of Public Health, University of Copenhagen, Copenhagen, Denmark; Department of Health Science and Technology, Physical Activity and Human Performance group, SMI, Aalborg University, Aalborg, Denmark

**Keywords:** Sickness absence, Disability pension, Retirement, Occupational health, Ageing population

## Abstract

**Background:**

As average life span increases, elderly will account for an increasing proportion of the total population in most parts of the world. Thus, initiatives to retain older workers at the labor market are becoming increasingly important. This study will investigate the influence of physical and psychosocial work environment throughout working life and physical and cognitive capacity in midlife on labor market attachment among older workers.

**Methods/Design:**

Approximately 5000 participants (aged 50–60 years) from the Copenhagen Aging and Midlife Biobank (CAMB) will be followed prospectively in a national register (DREAM), containing information on a week-to-week basis about social transfer payments for about 5 million Danish residents. Using Cox regression, we will model the risk of long-term sickness absence, disability pension, early retirement and unemployment within a 4 to 6 year period from the baseline measurement as a function of the following predictors: 1) physical work demands throughout working life, 2) psychosocial working conditions throughout working life, 3) physical capacity in midlife, 4) cognitive capacity in midlife. Estimates will be adjusted for age, sex, lifestyle, socioeconomic position, chronic disease and long-term sickness absence prior to baseline.

**Discussion:**

The project will generate new knowledge on risk factors for loss of labor market attachment. The results will potentially contribute in identifying factors that could be targeted in future interventions for maintaining a longer and healthier working life among older workers.

## Background

In the future, elderly will account for an increasing proportion of the total population in Denmark and in the European Union [1]. The potential costs associated with a growing elderly population has motivated Denmark and other EU countries to develop and implement initiatives to encourage older workers to stay longer at the labor market [2]. Until a few years ago, Danish workers had the possibility of early retirement at the age 60. With the adoption of the early retirement reform in 2011, this limit will gradually increase to 64 years (for persons born in 1959 and onwards) and longer working lives will now be expected of all. Similar trends are seen across many European countries. A long, healthy and productive working life is therefore a political priority, and in recent years the focus of several European countries have been to create a better framework for keeping older workers on the labor market [3].

However, several factors could potentially threaten the possibility of keeping a larger proportion of older workers at the labor market. Work requirements will remain high or even increase in many industries, while physical capacity naturally decreases with age [4]. This can lead to reduced work ability [5], difficulties to cope with the requirements of the work, and thus increased risk of long-term sickness absence, unemployment and permanent drop-out of the labor market. For example, muscle strength decreases by an average of 1–2 % per year from the age of 30 [6]. Consequently, workers between 50 and 60 years of age will on average have lost over a third of their original muscle strength. Similarly, age-related decline in cognitive abilities have been reported (including decline in memory, reasoning, phonetic and semantic functions) [7], although older workers seem to partly compensate for this cognitive decline [8]. The variation of physical and cognitive resources also increases with age and a significant proportion of older workers may therefore lack the resources to cope with the demands of the work [4].

The Danish Work Environment Cohort Study showed that two thirds of the 50–59-year-old workers plan to withdraw from the labor market before statutory retirement age that will gradually increase from 65 to 67 [9]. Not surprisingly, higher physical job demands were associated with earlier planned withdrawal from the labor market. Both psychosocial and physical working environment play an important role in this context. Many older workers with physical demanding jobs are uncertain whether they will be physically able to perform their work until they are 67. However, a portion of the older workers would chose a later retirement if reduced working hours was a possibility or if they had greater influence on the planning of working hours [9]. Influence at work is an important part of the psychosocial work environment [10] which may also have substantial consequences for the physical work environment. A review of 8 prospective studies showed that high physical work demands, high work pressure, low job satisfaction, poor health and lack of physical activity in leisure time is associated with increased risk of early retirement [11]. In addition, a Danish study showed that lack of recognition and poor possibilities for developing new skills among older male workers are strongly associated with retirement planning [12]. Hence, to increase the proportion of older workers who stay on the labor market, knowledge of both physical and psychosocial risk factors and protective factors for labor market attachment is needed.

Prevention of long-term sickness absence is a key factor to retain healthy and productive older workers at the labor market. Sickness absence reflects the complex interaction of health and work characteristics [13] and can be a consequence of the scenario where work requirements exceeds individual capacity. Sickness absence predicts several work related outcomes such as unemployment [14, 15] and future disability pension among older workers [16, 17]. Importantly, previous research showed that older workers less often are on sick leave compared with younger workers, but the duration of sickness absence are generally longer [18–20]. Several risk factors in the physical and psychosocial work environment for sickness absence have previously been identified. Specifically, exposure to physical workloads, such as lifting, bending or twisting of the back, squatting and kneeling, standing and repetitive arm/hand movements, has been identified as risk factors for sickness absence in the general working population [21–23]. Importantly, exposure to several of these single risk factors seems to have even larger consequences, as illustrated by Andersen and co-workers who reported that a higher number of combined physical workloads were associated with progressively higher risk for long-term sickness absence [23]. Of the psychosocial factors, especially low job control and decision authority are key factors associated with sickness absence [24–27]. Thus, to effectively prevent sickness absence, unemployment and disability retirement among older workers, a better understanding of lifelong exposure to several physical and psychosocial work factors is needed.

### Aim

The aim of the study is - through a 4–6 year prospective register based follow-up study on the Copenhagen Aging and Midlife Biobank (CAMB) – to investigate the influence of physical and psychosocial work demands throughout life and of physical and cognitive capacity in mid-life on labor market attachment among older workers (in terms of risk of long-term sickness absence, disability pension, early retirement and unemployment). The present study will answer the following research questions:

- Are physical and psychosocial working conditions throughout life associated with risk of long-term sickness absence, disability pension, early retirement and unemployment?
- Are high physical and cognitive capacities in midlife associated with lower risk of long-term sickness absence, disability pension, early retirement and unemployment?

### Hypotheses

We will test the following hypotheses:

- High physical work demands throughout life are associated with increased risk of long-term sickness absence, disability pension, early retirement and unemployment.
- Adverse psychosocial working conditions throughout life are associated with increased risk of long-term sickness absence, disability pension, early retirement and unemployment.
- Low physical capacity in midlife is associated with an increased risk of long-term sickness absence, disability pension, early retirement and unemployment, whereas high physical capacity is associated with a decreased risk.
- Low cognitive capacity in midlife is associated with an increased risk, of long-term sickness absence, disability pension, early retirement and unemployment, whereas high cognitive capacity is associated with a decreased risk.

## Methods/Design

### Study design

The project is a 4 to 6 year prospective follow-up study. Using participant’s social security number, we will link the CAMB database, containing information on work environment and health, with the Danish Register for Evaluation of Marginalization (DREAM), containing information on all transfer payments [28].

### Study population

In 2009–2011 the Copenhagen Aging and Midlife Biobank (CAMB) data collection was conducted by researchers from Department of Public Health, University of Copenhagen, in collaboration with the National Research Centre for the Working Environment (NRCWE) [29]. The CAMB database contains data on biological, psychological and social factors for persons between 50 and 60 years of age from the merging of three established cohorts: The Metropolit Cohort [30], The Copenhagen Perinatal Cohort [31] and the Danish Longitudinal Study on Work, Unemployment, and Health [32]. A total of 17,937 individuals were invited, and 7190 responded to the questionnaire of which 5575 attended the clinical examination. The data collection included measures of physical and cognitive resources and questionnaires on physical and psychosocial work environment and health. CAMB participants who were not employed will be excluded from the analysis, i.e. those on disability pension and being unemployed, which will yield a study sample of approximately 5000 working individuals at baseline. More than 80 % of these participated in the various physical tests (except for the aerobic capacity test that was conducted on approximately 1000 individuals).

### Predictor variables

The following predictor variables will be included in the analyses:

#### Physical work demands

The physical work demands throughout the working life will be evaluated from the CAMB questionnaire by a general question on physical exposure during work: “Looking back on your entire working life: For how many years of your working life have you had…, 1) mostly sedentary work without physical strain?, 2) mostly standing or walking work without major physical strain?, 3) mostly standing or walking work with some lifting and carrying?, 4) mostly heavy, fast or physically demanding work?”. These four response categories were based on a question from the Copenhagen Male Study [33]. For each response category respondents listed the number of years of working life (cumulative exposure assessment) with the specific effort level [34]. Subsequently, the data of exposure years in each of the 4 categories will be recoded to a number between 0 and 100, where 0 indicates that all exposure years belong to category 1 (seated work) and 100 indicates that all exposure years belong to category 4 (very hard work), and anything in between will be linearly scaled. Finally, categories will be defined as low physical work demands (0–24.99), moderate physical work demands (25–49.99), high physical work demands (50–74.99) and very high physical work demands (75–100).

In addition, participants replied to questions on risk factors for musculoskeletal disorders or other health hazards: “In your current or previous job are/were you often exposed to the following in your daily work (several times a week or more)…1) noise so loud that you must raise your voice to talk to other people?, 2) hand tools vibrations?, 3) lift or move heavy things or persons?, 4) pull or push heavy burdens?, 5) work in stooping posture without leaning on hands or arms?, 6) work in which you have to twist or bend your back several times per hour?, 7) work where you repeat the same movements several times per minute during a large part of the working hours?, 8) dust? (cement, demolitions, mineral fibers, wood, animals or plants), 9) toxic substances?, 10) welding smoke?, and 11) diesel fumes?”. The following response categories were available: “no”, “yes”; “if yes, indicate number of years”.

#### Psychosocial working conditions

Psychosocial working conditions throughout the working life was assessed in CAMB by questions derived and modified from the Copenhagen Psychosocial Questionnaire [35]: “Looking back on your entire working life: 1) how often did you not have time to complete all your work tasks?, 2) did you have a large degree of influence concerning your work?, 3) did you have to relate to other people’s personal problems as part of your work?, 4) did your work require you to make difficult decisions?, 5) did you have to work very fast?, 6) was there a good atmosphere between you and your colleagues?, 7) did your colleagues talk with you about how well you carry out your work?, 8) did your nearest superior talk with you about how well you carry out your work?, 9) were contradictory demands placed on you at work?, 10) did you know exactly which areas were your responsibilities?, 11) did you have the possibility of learning new things through your work?, and 12) was your work recognized and appreciated by the management?”. The response category for question 1–8 was: 1) “always”, 2) “often”, 3) “sometimes”, 4) “seldom”, 5) “never/hardly never”. The response category for question 9–12 was: 1) “to a very large extent”, 2) “to a large extent”, 3) “somewhat”, 4) “to a small extent”, 5) “to a very small extent”.

#### Physical capacity

Physical capacity was assessed through physical tests described in detail elsewhere [36]. The physical tests included measurement of reaction time, postural balance, lung functioning, aerobic capacity, flexibility, jump height, sit-to-stand test, static muscle strength of the back and abdominal muscles, as well as static and explosive muscle strength of the hand flexor muscles. For the physical tests we will define low and high capacity as 1 standard deviation below and above average, respectively, for each gender separately.

#### Cognitive capacity

Cognitive capacity was assessed with the Intelligenz-Struktur-Test 2000 R (I-S-T 2000R), which provides a global measure of cognitive function [37]. The CAMB version of the I-S-T 2000R consists of sentence completion (19 items), verbal analogies (20 items), and number series (20 items), and the scores on the three subtests are combined to a total score with a 0 to 59 range. We will use the total score in the primary analysis, because the subscores are moderately to highly correlated. For the cognitive tests we will define low and high capacity as 1 standard deviation below and above average, respectively, for each gender separately.

#### Self-rated function

Self-rated function was assessed with selected questions from MFI (Multidimensional Fatigue Inventory), SF36 along with questions related to physical function from the CAMB questionnaire. We will compare self-rated function with the objectively measured physical and cognitive capacities (described above) to identify the strongest predictors of labor market attachment.

### Outcome variables

Information on long-term sickness absence was derived from a Danish register of social transfer payments (DREAM), and linked to the CAMB cohort via the unique social security number which is given to all Danish citizens at birth. The DREAM register contains information on all types of transfer payments (including sickness, early retirement, government education, unemployment benefits etc.) and other basic personal data on about 5 million Danish residents on a weekly basis [38]. The outcome variables are labor market attachment to varying degrees:

- Long-term sickness absence – sickness absence of at least 5 consecutive weeks.
- Disability pension.
- Early retirement.
- Unemployment.

### Covariates

Covariates include age, sex, lifestyle factors (BMI, smoking, physical activity), socioeconomic position (from CAMB database), chronic disease (from CAMB questionnaire) and long-term sickness absence at baseline (from DREAM register).

### Statistics

The prospective analysis will be carried out with Cox regression in SAS using the PHREG procedure [39, 40]. Time to event is defined as the number of days from baseline to the outcome in the DREAM register within a 4–6 year period from the baseline measurement. When individuals have an onset of long-term sickness absence, disability pension, early retirement or unemployment within the follow-up period, the survival times will be non-censored and referred to as event times. The analyses will include only those who were employed at baseline (approximately 5.000 individuals). The analyses on long-term sickness absence will be censored for death or any other form of permanent dropout from the labor market in the follow-up period (i.e. disability pension, early retirement or retirement). Results will be reported as HR’s with 95 % CIs.

### Additional data analyses

Self-reported physical demands at work are supplemented by data from a job exposure matrix “the Lower Body JEM”, [41] which have previously been used in the CAMB data set in a Ph.D. project on the association between physical exposures in working life and physical function in midlife [42]. The Lower Body JEM is based on expert judgments of physical exposures associated with risk of osteoarthritis in the lower limb: sitting, standing/walking, whole-body vibration, kneeling, and lifting (weight and number of heavy lifts) [42]. Job titles that include physical exposures were grouped in 121 so-called “homologue Exposure Group” (HEGs) in the JEM. The division and assessment of the daily load in HEGs was carried out by a panel of five specialists in occupational medicine and a median load was subsequently calculated [43, 44]. The CAMB questionnaire includes information about job history and all job titles have been recoded to DISCO job titles (the Danish version of the international Classification of job titles; DISCO-88, Statistics Denmark) [42]. The Lower Body JEM is also based on DISCO job titles and thus, an individual cumulative load can be calculated for each of the physical exposures. Finally, the cumulative load can be converted to standardized load-year and further analyzed [42].

### Power calculation

Based on extracts from the DREAM database of people aged 50–59 years who are working at baseline (*n* = 757,226), the following incidence rates (new cases) were observed over a 3-year period (from 2009 to 2011):

- One period of long term-sickness absence (at least 5 consecutive weeks): 11.7 %.
- Disability pension: 2.9 %.
- Early retirement pension: 6.7 %.
- Unemployment: 13.0 %.

**Fig. 1:**
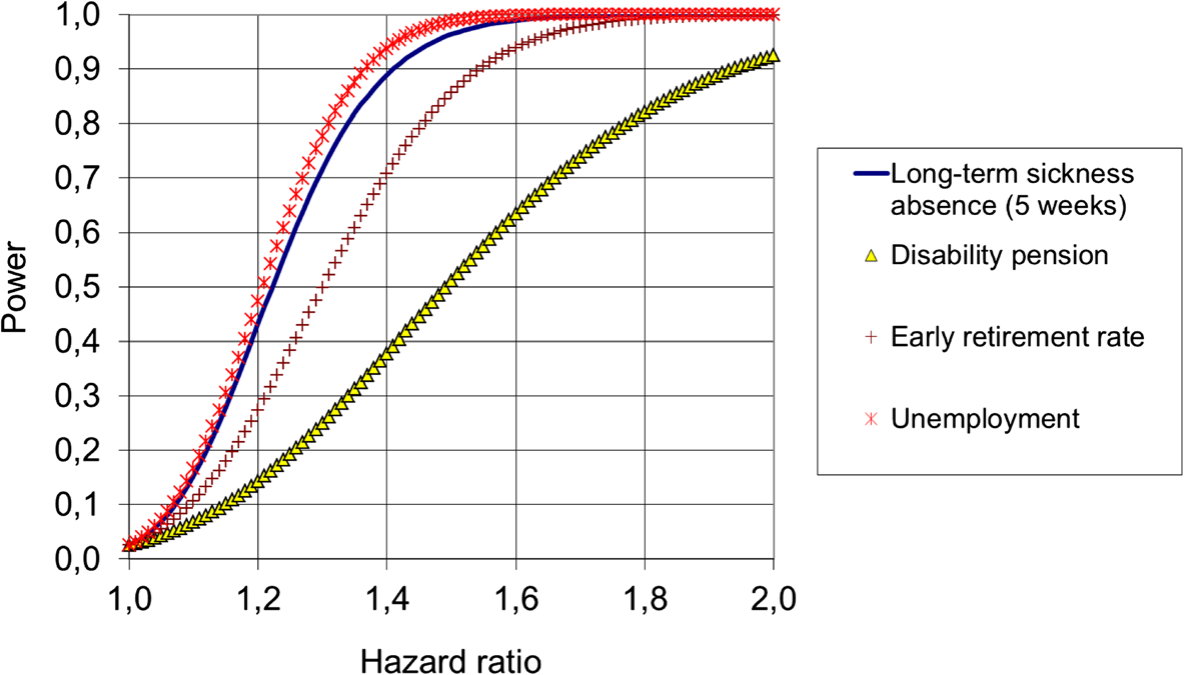
The probability (power) to detect a difference between people in the upper vs. lower tertile at a given scale as a function of hazard ratio

Power calculations are based on these incidence rates. Calculations assume dual hypothesis testing, and a significance level of 0.05. The probability (power) to detect a difference between people in the upper vs. lower tertile at a given scale is shown in Fig. 1, as a function of hazard ratio. For example, if the true hazard ratio for 5 consecutive weeks of sickness absence is 1.4, we will have an 89 % chance to demonstrate this difference. As depicted in Fig. 1, compared with long-term sickness absence, statistical power is less for early retirement and disability pension, whereas we have slightly more power with unemployment.

### Scientific dissemination

Four articles on the results of the project will be submitted to international journals with peer review: 1) one article focusing on the physical work demands and risk of long-term sickness absence, disability pension, early retirement and unemployment, 2) one article focusing on psychosocial working conditions and risk of long-term sickness absence, disability pension, early retirement and unemployment, 3) one article focusing on physical capacity in midlife and risk of long-term sickness absence, disability pension, early retirement and unemployment, 4) one article focusing on cognitive capacity in midlife and risk of long-term sickness absence, disability pension, early retirement and unemployment.

## Discussion

### Scientific novelty

The project utilizes the unique large dataset in the CAMB study to implement a comprehensive investigation of the relationship of working environment throughout life and physical and cognitive capacity in midlife with the risk of dropping out of the labor market. The project will contribute to knowledge on risk factors and protective factors for labor market attachment. The results may contribute in identifying factors that should be targeted in future interventions for maintaining a longer and healthier working life among older workers. This includes identifying individuals who especially need preventive action to increase their possibility for remaining in work until retirement.

### Strengths and limitations

The physical and psychosocial work environment throughout life was retrospectively assessed by self-reports at mid-life and not continuously assessed throughout the lives of the participants. The type and exposure time of the physical workloads and the different dimensions of the psychosocial work environment could therefore be prone to potential bias and thus less accuracy (especially recall bias). For example, information of physical workload assessed through questionnaire surveys depends on participants memory, understanding and interpretation [45]. In the present study, this may cause wider CIs of the risk estimates, and thus increase the probability for a type II error. In addition, questionnaire information on several physical behaviours seems to be systematically biased by factors such as disease, and socioeconomic and demographical status [46, 47], which could lead to an increased probability of a type I error. However, this probability will be reduced in the present study by adjusting for several factors that potentially could lead to self-reporting bias (e.g. chronic disease and socioeconomic position). However, the results of the present study should be interpreted within the limitations mentioned above.

A strength of the project is that there are tests of physical resources and test of cognitive function on a large group of workers at 50–60 years of age, as well as standardized questions about the physical and psychosocial work environment that will be linked prospectively to the DREAM register. The DREAM register has high reliability, because all transfer payments are systematically recorded and employers have a financial incentive to report long-term sick leave. It is a weakness of the study that the working environment is not objectively measured, and as with other surveys, a risk of reporting bias exists. To address this weakness, we will therefore, in addition to including a questionnaire on the work environment, also test the model with the Lower Body JEM [41, 42] as a proxy measure of physical exposures throughout working life.

The strength of the CAMB study compared with previous studies is that physical and cognitive capacities have been objectively measured rather than self-reported through questionnaire surveys. Obtaining objective measurements is much more challenging than using questionnaires alone, but the measurements are more precise. As an example, there is only a weak to moderate correlation between self-reported muscle strength (questionnaire) and objectively measured muscle strength (*r* = 0.30 to 0.51, i.e., explained variation between 9 and 26 %) [48, 49]. In addition, in the CAMB project we minimize the “common methods variance”, where individuals answering questions about their own physical and cognitive resources may be affected by their own perception of the work environment. For example, a person with physically demanding work perceive and even report own resources differently than a person with a sedentary job. In the CAMB study a large database has been established where tests of physical and cognitive resources are available for approximately 5000 people between 50 and 60 years. By coupling the CAMB study to record information about labor market attachment, it is possible to overcome some of the weaknesses in studies based on exclusive use of questionnaires.
